# A Prospective Study Investigating the Health Outcomes of Bitches Neutered Prepubertally or Post-Pubertally

**DOI:** 10.3390/ani15020167

**Published:** 2025-01-10

**Authors:** Rachel Moxon, Sarah L. Freeman, Richard M. Payne, Sandra Corr, Gary C. W. England

**Affiliations:** 1 Canine Science, Guide Dogs National Centre, Banbury Road, Leamington Spa, Warwickshire CV33 9WF, UK; rachel.moxon@guidedogs.org.uk; 2 School of Veterinary Medicine and Science, University of Nottingham, College Road, Sutton Bonington, Leicestershire LE12 5RD, UK; richard.payne@nottingham.ac.uk (R.M.P.); gary.england@nottingham.ac.uk (G.C.W.E.); 3 School of Biodiversity, One Health and Veterinary Medicine, College of Medical, Veterinary and Life Sciences, University of Glasgow, Bearsden Road, Glasgow G61 1QH, UK; sandra.corr@glasgow.ac.uk

**Keywords:** dog, bitch, neuter, puberty, disease, health

## Abstract

There are scant studies of associations between the pubertal status at neutering and subsequent health outcomes of female dogs. This study examined health data for Labrador and Golden Retriever crossbreed bitches neutered by ovariohysterectomy either before (PrePN, *n* = 155) or after (PostPN, *n* = 151) puberty using a prospective study design. Data were extracted from an electronic database containing detailed health records for all bitches. Cruciate ligament disease occurred in significantly more PrePN (*n* = 11) than PostPN bitches (*n* = 1; Yates Chi-square = 6.784, D.F. = 1, *p* = 0.009), and PrePN bitches had lower probabilities of remaining free from osteoarthritis (χ^2^ = 5.777, D.F. = 1, *p* = 0.016). Additionally, PrePN bitches were diagnosed with atopic dermatitis (PrePN: 3.9 ± 0.6 years, PostPN: 1.6 ± 0.3 years; Mann–Whitney U test = 90.5, *p* = 0.008) and perivulval dermatitis (PrePN: 4.7 ± 0.9 years, PostPN: 0.8 ± 0.1 years; Mann–Whitney U test = 40.0, *p* = 0.002) at significantly older ages and had lower probabilities of remaining free from otitis externa (χ^2^ = 7.090, D.F. = 1, *p* = 0.008). No associations were identified between the pubertal status at neutering and the incidence of any urogenital disease, including urinary incontinence, which was diagnosed in a small number of bitches (one PrePN and six PostPN). The results suggest that prepubertal neutering may have a detrimental effect on some future musculoskeletal and immune diseases in bitches of these crossbreeds, providing important information to support neutering policies and to help maintain optimal dog welfare.

## Simple Summary

Very few studies have been identified that have investigated associations between neutering before or after known puberty on later health outcomes in female dogs. This study examined data on the long-term health of 306 Labrador and Golden Retriever crossbreed bitches neutered before (*n* = 155) or after (*n* = 151) puberty in a UK assistance dog programme. Data were gathered from bitches’ detailed electronic health records to a maximum of 11.5 years of age. Associations were identified between neutering before or after puberty and musculoskeletal disease and immune diseases. Bitches neutered before puberty had significantly more cases of cruciate disease and were diagnosed with osteoarthritis at younger ages than bitches neutered after puberty. Bitches neutered before puberty were older at the first diagnoses of atopy and of perivulval dermatitis, and had lower probabilities of remaining free from otitis externa. Unexpectedly, no associations were identified between the pubertal status at neutering and the incidence of any urogenital disease. The results suggest that neutering before puberty may have a detrimental effect on some future musculoskeletal and immune diseases in female dogs of these crossbreeds. This information provides important information to support neutering policies and to help maintain optimal dog welfare.

## 1. Introduction

The timing of neutering, most frequently related to the age at neutering, has been suggested to impact the health outcomes of female dogs [1,2,3,4,5,6,7,8,9]. However, the evidence describing the impacts on health is not consistent across diseases. This is further complicated by breed differences, and variations between studies in terms of populations and methodology [4,5,10].

The lack of agreement within the literature is reflected in veterinarians’ opinions with regard the effect of the timing of neutering on bitch health. A survey of 723 UK veterinarians [11] revealed that 42% believed neutering before or after the first oestrus had no impact on the development of future health issues in bitches. However, 17% believed that prepubertal neutering was associated with more health issues, whilst 23% believed that post-pubertal neutering was associated with more health issues. This disagreement is unsurprising as there is a lack of evidence regarding the effects of neutering timing in relation to puberty on female dog health, with questions raised regarding the reliability of some study methodologies and findings [10]. Dogs can reach sexual maturity as early as six to seven months of age [12,13,14,15]. Some studies that have purported to examine the outcomes of neutering bitches in relation to puberty have in fact neutered bitches at different ages and inferred findings related to puberty [6,16]. This makes interpreting the findings of studies related to the impacts of neutering in relation to known puberty more difficult. A recent scoping review of the literature examining the health impacts of neutering identified only six studies that considered whether bitches were surgically neutered before or after puberty [10]. None of these studies investigated common diseases associated with neutering, including atopy, musculoskeletal disease, or obesity.

This study aimed to investigate the effects of the timing of neutering in relation to puberty (rather than the age at neutering) on the future health outcomes of bitches from an assistance dog programme. This will provide important information to support decision making regarding whether to neuter female dogs before or after puberty.

## 2. Materials and Methods

### 2.1. Study Design

A prospective study was undertaken using 306 bitches born between 22 February 2012 and 9 August 2015 in an assistance dog programme. Bitches were allocated to groups to be neutered either prepubertally (at six months of age, before their first oestrus) or post-pubertally (after their first oestrus), as previously described [17]. Data were gathered to examine the health of bitches in the study from six months of age to 15 September 2023.

### 2.2. Study Setting

Within the assistance dog programme, dogs are placed into volunteer homes with puppy raisers between seven and eight weeks of age. During the puppy raising stage, dogs are neutered before entering formal assistance dog training at approximately 14 months of age. The standard practice is for female dogs to be neutered after their first oestrus. During training stages, dogs are housed in kennels or with volunteer boarders in their homes. Dogs qualify as assistance dogs between two and three years of age. Dogs in the programme are managed under similar conditions, and all are fed the same commercially available extruded dry diet from weaning. The assistance dog organisation is continually responsible for the dogs’ health and any associated veterinary investigations and treatments, including when dogs are working with assistance dog owners. A team of Dog Health and Wellbeing Specialists oversees the health of all dogs. All health problems and consultations are recorded in the organisation’s electronic database and are coded as health items. Health items are only coded for a disease once a diagnosis is confirmed using appropriate diagnostic tools in consultation with a veterinarian or specialist, i.e., radiograph or computed tomography for elbow dysplasia, and following referral and work-up with a specialist dermatologist for atopy. The database is searchable, enabling the identification and extraction of data for any dogs with a particular health code. Additionally, each dog’s whole health records can be extracted to allow manual searches of all text entries. Dogs that do not qualify as an assistance dog, that qualify but then are withdrawn from working before planned retirement, or that retire are rehomed with either the assistance dog owner or, most likely, new owners (rehomers). Following rehoming, the responsibility for the dog’s health is relinquished and health data cease to be gathered. Rehomers are provided with a letter requesting that they inform the organisation should the dog be diagnosed with certain health conditions. When dogs die, their status in the database is changed to ‘deceased’ and a health record is added to note the date and cause of death, where known.

### 2.3. Study Animals

Bitches were from five different Labrador/Golden Retriever crossbreeds and were allocated into two groups: neutered prepubertally (at six months of age—PrePN, *n* = 155) or post-pubertally (after their first oestrus—PostPN, *n* = 151; Table 1). All first-generation backcross bitches were grouped into one breed group for analysis. All bitches were neutered by ovariohysterectomy at one of four UK veterinary practices following a standardised neutering protocol [17]. The mean age at neutering was 189.4 ± 0.4 days for PrePN bitches and 387.8 ± 3.2 days for PostPN bitches. PostPN bitches were neutered between 20 and 194 days after oestrus.

### 2.4. Variables and Data Sources

#### 2.4.1. Diseases

Diseases included in the study were those determined to be of importance to the assistance dog organisation. These were identified by examining data on withdrawals from the programme due to health and advised by the organisation’s Chief Veterinary Officer and Breeding Dog Health and Screening Specialist, and/or diseases that had been cited as being impacted by the timing of neutering in the scientific literature. Diseases were also included if they had been identified in two previous projects (including a survey of practising veterinarians) as potentially impacted by neutering timing [11,18] (Supplementary Material S1). Outcome variables were (1) being affected with the disease and (2) age at first diagnosis.

The complete list of health items (*n* = 2249) that are used within the organisation to code the disease, diagnostic tests, and routine treatments was obtained. This was reviewed and all codes pertaining to each of the diseases of interest were retained. Health code searches were run to generate reports in Excel for all dogs with each health code that were born between 22 February 2012 and 9 August 2015. Data for all health codes that related to one disease were combined onto one spreadsheet (Microsoft Excel) (e.g., for atopic dermatitis (atopy), the health codes included were ‘acute atopic conjunctivitis’; ‘allergic skin disease’; ‘atopic [allergic] otitis’; ‘atopic conjunctivitis’; and ‘atopic dermatitis’) (Supplementary Material S2). The date of the first diagnosis was extracted and used to calculate the age at first diagnosis. Only new diagnoses that occurred after six months of age were included; diagnoses before six months of age were ignored unless there was a subsequent new episode after six months of age. For being overweight, the date of the first health record where the dog was recorded as being 5% or more over the optimum weight was used. Adult body condition scores (BCSs) were recorded as the highest BCS for each bitch at ≥2.5 years of age along with the date of the BCS. To ensure that no data were missed, complete health records for each bitch were extracted and manually searched to identify any cases where a disease had been described in the veterinary health notes but had not been coded in the database. Health data were correct up to 15 September 2023. The date of the last available health record was recorded as either the last health record entry for bitches that had left the programme by the time of data collection or as 15 September 2023 for those that remained working.

#### 2.4.2. Withdrawals from Working for Health Reasons and Causes of Death

The current status (working, withdrawn, transferred, or deceased) of all bitches was extracted from the database on 15 September 2023. Any bitches that withdrew from working before reaching retirement at the time of data collation had their withdrawal records examined. Reasons for health-related withdrawals were extracted. For all bitches with a status of ‘deceased’, the date of death was extracted and used to calculate the age at death. Health records were examined to determine the cause of death. Outcome variables were the number of bitches that were withdrawn from working for health reasons, the number that had died, and age at death.

### 2.5. Bias and Study Size

Random effects and confounding factors were included in the statistical analysis using Cox regression models when examining the main effect of the trial group on the dependent variables (diseases). Bitches in this study were of similar breeds, and breeds were not different between trial groups. However, the breed was included to control for the potential effect on other variables. Where relevant to the disease being studied, data generated for a previous study relating to physical development [19], such as the change in height for musculoskeletal disease and data on vulval appearance and development for urogenital disease, were included in the models.

The study size was limited by the available cohort of bitches produced during the study recruitment period and that were placed in puppy raising within a suitable distance to travel to one of four national veterinary practices for neutering surgery. Values of alpha were determined using G*Power Version 3.1.9.7 (https://www.psychologie.hhu.de/ (accessed on 8 June 2022)) for each analysis (see Supplementary Material S3) to minimise the likelihood of Type I and II errors and in order to use values of alpha appropriate for the sample size and size of effect that would be expected to be significant. The means, SEMs, and confidence intervals (CIs) were reported, where appropriate.

### 2.6. Quantitative Variables and Statistical Methods

The survival analysis was conducted using SPSS version 28 (IBM Corp, Armonk, NY, USA), Chi-square tests, Mann–Whitney U tests and t-tests were run in XLStat (XLStat2016, Addinsoft, New York City, NY, USA).

#### 2.6.1. Diseases

Disease data were grouped into one of five categories: musculoskeletal diseases, malignant neoplasia, urogenital diseases and pseudopregnancy, immune disease, and ‘other’ diseases. PrePN bitches were neutered before puberty; therefore, they were not able to experience a pseudopregnancy. Pseudopregnancy was reported under urogenital disease, but comparisons were not made between groups and all statistical analyses of urogenital diseases excluded pseudopregnancy. For urogenital diseases, descriptive data were presented for the number of bitches affected with or clear from each disease that had different vulval appearances and development data available from assessments at 17 months of age [19].

The number of bitches diagnosed with each disease, the number that had at least one disease from each category, and the number of bitches that had 0, 1, or 2 diseases in each category were compared between trial groups using Chi-square tests when the numbers were sufficient for analysis. Values of alpha were 4 or 5%, and beta was 5% for all analyses with effective sample sizes between 39 and 324 dogs (see Supplementary Material S3). The number of PostPN bitches diagnosed with a disease before neutering was indicated for all diseases where this was observed. The percentage of bitches that were classified as obese based on having an adult BCS of 7, 8, or 9 was reported and compared between groups using Chi-square analysis. Alpha and beta were both 5%, with an effect size of 0.270.

The disease incidence rate was determined for PrePN and PostPN groups for diseases with more than 10 bitches affected. The denominator was the total number of dog-days in the study (the number of days of health record data available from six months of age for all bitches in the group). The total number of episodes of disease diagnosed was the numerator. The rate was multiplied by 1000 to provide a disease incidence rate per 1000 dog-days. The incidence rate ratio (IRR) was calculated using the formula: Disease Incidence Ratio = [PrePN disease incidence rate/1000 dog-days]/[PostPN disease incidence rate/1000 dog-days]. The 95% CIs (for alpha = 5%) were calculated using the following formula:

$$95\%\;\mathrm{CI}\;\mathrm{for}\;IRR=\exp\{\ln\left(IRR\right)\pm ZySD\left[\ln\left(IRR\right)\right]\}$$

where Zy is 1.96, the critical z-score value for a 95 percent CI [20].

For diseases with more than 10 bitches affected, the mean age at the first diagnosis was compared between trial groups using *t*-tests or Mann–Whitney tests, depending on the data distribution. Alpha and beta were determined using sensitivity analysis. Values of alpha were between 9% and 13% and beta was 5% for all analyses, with effect sizes between 0.572 and 2.124 (see Supplementary Material S3). For musculoskeletal diseases with more than 10 bitches affected, data for the change in height between six and 17 months of age [19] were examined to identify any differences in growth between bitches that were and were not affected with disease using Mann–Whitney tests. Alpha and beta were 9% and 5% for all analyses, with effect sizes between 0.394 and 0.400 (see Supplementary Material S3). For urogenital diseases, data for the vulval appearance and development were examined to identify associations with urogenital diseases using Chi-square tests. Data for the vulval appearance at 17 months of age were grouped into ‘normal’ and ‘not normal’, data for the dorsal fold coverage were grouped as ≤20% and >20%, and data for overall vulval growth were grouped as ‘smaller or the same’ and ‘larger’ for the analysis. Alpha and beta were 4% and 5% for all analyses, with effect sizes between 0.396 and 1.124 (see Supplementary Material S3).

Kaplan–Meier survival curves and Cox regression analysis were performed to examine differences between PrePN and PostPN bitches in remaining free from disease. Differences between curves were examined using log rank tests. Cox regression analysis was used for diseases with more than 10 bitches affected per predictor variable [21]. Risk factors included in all models were the breed and trial group. The models for musculoskeletal diseases included data for the change in height between six and 17 months of age. The models for urogenital diseases included data on the vulval appearance (normal, recessed, and/or juvenile), percentage dorsal fold coverage at 17 months of age, and vulval growth (larger, smaller, or no change) between six and 17 months of age. The models for overweight/obesity included BCSs at six and 17 months of age. Statistical power for the Cox regression analysis was investigated post hoc using MedCalc Statistical Software version 19.2.6 (MedCalc Software bv, Ostend, Belgium; 2020), https://www.medcalc.org (accessed on 22 November 2023). However, the power was so low due to the small sample sizes that these analyses were not reported; in order to visualise the data, Kaplan–Meier survival curves were presented.

#### 2.6.2. Withdrawals from Working for Health Reasons and Cause of Death

The number of bitches in each trial group that were withdrawn from working for health reasons and that had died were compared using Chi-square tests. A *t*-test was used to examine differences in the age at death between PrePN and PostPN bitches. For withdrawals from working, alpha and beta were both 5%, with an effective sample size of 195 dogs. For withdrawals from working for health reasons and the number of bitches that died, alpha and beta were 4% and 5%, with effective sample sizes of 95 and 236 dogs, respectively. For the age at death, alpha and beta were 10% and 5%, respectively, with an effective sample size of 32 dogs.

## 3. Results

### 3.1. Participants

Health data were available for bitches until the time they died or were withdrawn or retired from working and rehomed. At the time of data collation (15 September 2023), 82 bitches (33 PrePN and 49 PostPN) remained working as assistance dogs, 148 had been withdrawn and rehomed (10 with assistance dog owners), 37 had been retired and rehomed (25 with assistance dog owners), seven had been transferred to other working dog organisations, and 32 had died. Bitches were between 0.5 and 11.1 years of age when they left the programme and bitches that remained in the programme at the point of data collation were between 8.1 and 11.5 years of age. There was no significant difference in the amount of available data (years) between the PrePN and PostPN groups (Chi-square = 6.548; D.F. = 4, *p* = 0.162).

### 3.2. Diseases

The number and percentage of bitches diagnosed with each disease along with descriptive data for the age at diagnosis are presented in Supplementary Material S4. No multivariable Cox regression models are presented due to small numbers of bitches with each disease and inadequate power.

#### 3.2.1. Musculoskeletal Diseases

There were 82 bitches (47 PrePN and 35 PostPN) diagnosed with at least one musculoskeletal disease (Chi-square = 1.990, D.F. = 1, *p* = 0.158). There were no significant differences in the numbers of bitches diagnosed with zero (108 PrePN and 116 PostPN), one (31 PrePN and 30 PostPN) or two or more (16 PrePN and five PostPN) musculoskeletal diseases (Chi-square = 5.930, D.F. = 2, *p* = 0.052). The most frequently reported problem was forelimb lameness (29 PrePN and 25 PostPN [two diagnosed prior to neutering]; Chi-square = 0.244, D.F. = 1, *p* = 0.621), followed by osteoarthritis (19 PrePN and 11 PostPN; Chi-square = 2.139, D.F. = 1, *p* = 0.144). Cruciate ligament (CCL) disease occurred in significantly more PrePN (*n* = 11) than PostPN bitches (*n* = 1; Yates Chi-square = 6.784, D.F. = 1, *p* = 0.009). Elbow dysplasia (ED) (three PrePN and three PostPN [one diagnosed prior to neutering]), hip dysplasia (HD) (three PrePN and zero PostPN), patella luxation (one PrePN and zero PostPN), and other musculoskeletal diseases (three PrePN and one PostPN) occurred in too few bitches for statistical analysis. No bitches were diagnosed with osteochondritis dissecans (OCD) or juvenile osteochondrosis.

The IRR for CCL disease was 17.80 (95% CI = 2.36–134.25), for forelimb lameness was 1.27 (95% CI = 0.84–1.92), and for osteoarthritis was 1.92 (95% CI = 0.92–4.04).

**Figure 1:**
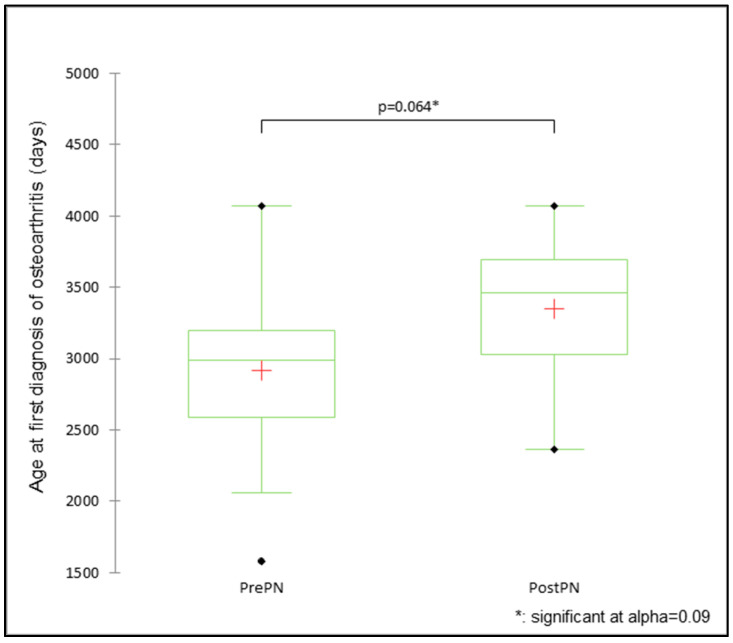
The mean, median, and range of days for the age at the first diagnosis of osteoarthritis for bitches neutered prepubertally (PrePN, *n* = 18) or post-pubertally (PostPN, *n* = 11).

The mean age at the first diagnosis was not significantly different between PrePN and PostPN bitches for forelimb lameness (PrePN 4.6 ± 0.5 years, PostPN 3.5 ± 0.5 years; Mann–Whitney U test = 451.5, *p* = 0.125). There was a significant difference in the age at the diagnosis of osteoarthritis (PrePN 8.0 ± 0.4 years, PostPN 9.2 ± 0.4 years; t = −1.935, *p* = 0.064; Figure 1) although effect size was −0.863, which was smaller than that determined by the power analysis (1.345). CCL disease and forelimb lameness were diagnosed multiple times for some bitches throughout their available health history. There were 17 CCL disease episodes in total (16 PrePN and one PostPN) and 90 episodes of forelimb lameness (48 PrePN and 42 PostPN).

There was no significant difference in growth between six and 17 months of age between bitches diagnosed with or unaffected by CCL disease (affected 5.64 ± 1.09 cm, unaffected 5.45 ± 0.19 cm; Mann–Whitney U test = 722.0, *p* = 0.811), forelimb lameness (affected 5.71 ± 0.45 cm, unaffected 5.42 ± 0.20 cm; Mann–Whitney U test = 3431.0, *p* = 0.471), osteoarthritis (affected 5.11 ± 0.59 cm, unaffected 5.50 ± 0.19 cm; Mann–Whitney U test = 2117.0, *p* = 0.556), or at least one musculoskeletal disease (affected 5.48 ± 0.35 cm, unaffected 5.47 ± 0.22 cm; Mann–Whitney U test = 4836.5, *p* = 0.889).

**Figure 2:**
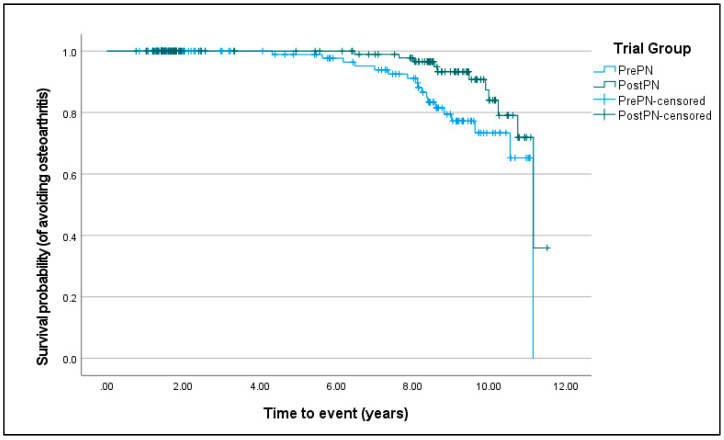
Kaplan–Meier curves of the survival probability (from having osteoarthritis) for bitches neutered before puberty (PrePN, blue) or after puberty (PostPN, green). Censored values (+) indicate the time that bitches left the programme.

**Figure 3:**
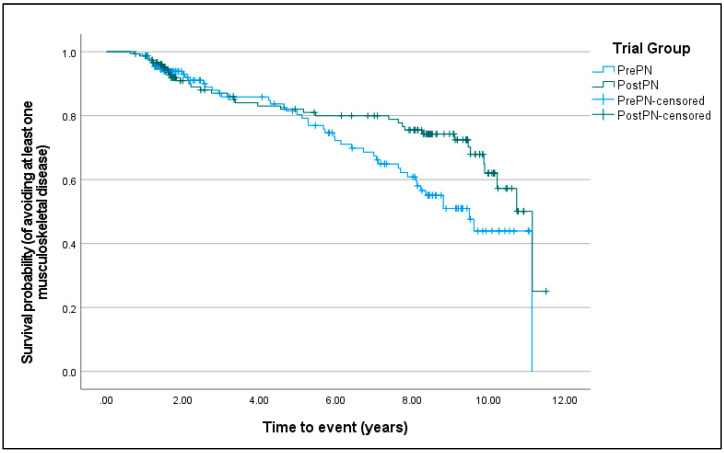
Kaplan–Meier curves of the survival probability (from having at least one musculoskeletal disease) for bitches neutered before puberty (PrePN, blue) or after puberty (PostPN, green). Censored values (+) indicate the time that bitches left the programme.

For forelimb lameness, Kaplan–Meier survival curves were not different between PrePN and PostPN bitches (χ^2^ = 0.970, D.F. = 1, *p* = 0.325). For osteoarthritis (χ^2^ = 5.777, D.F. = 1, *p* = 0.016; Figure 2) and having at least one musculoskeletal disease (χ^2^ = 5.142, D.F. = 1, *p* = 0.023; Figure 3), PostPN bitches had a significantly improved chance of avoiding disease development.

#### 3.2.2. Malignant Neoplasia

There were 38 bitches (21 PrePN and 17 PostPN) diagnosed with at least one malignant neoplasia (Chi-square = 0.369, D.F. = 1, *p* = 0.544). Only one bitch (PrePN) had two different types of malignant neoplasia diagnosed: a round cell tumour of the proximal tibia and neuroendocrine carcinoma with extensive metastasis. The most commonly reported problem was ‘other’ malignant neoplasia (12 PrePN and four PostPN; Chi-square = 4.004, D.F. = 1, *p* = 0.045), followed by mast cell tumours (MCTs) (four PrePN and seven PostPN; Yates Chi-square = 0.433, D.F. = 1, *p* = 0.510), although for both, there was no significant difference between the trial groups. Haemangiosarcoma (HSA) (one PrePN and two PostPN), lymphosarcoma (LSA) (two PrePN and two PostPN), melanocytic tumours (one PrePN and two PostPN), and squamous cell carcinoma (one PrePN and zero PostPN) occurred in too few bitches for statistical analysis. No bitches were diagnosed with adenocarcinoma, fibrosarcoma, mammary neoplasia, osteosarcoma, or transitional cell carcinoma.

The IRR for MCT was 0.87 (95% CI = 0.32–2.32) and for ‘other’ malignant neoplasias was 3.62 (95% CI = 1.18–11.09).

The mean age at the first diagnosis was not significantly different between PrePN and PostPN bitches for MCT (PrePN 8.4 ± 0.7 years, PostPN 8.2 ± 0.5 years; Mann–Whitney U test = 15.5, *p* = 0.824) or ‘other’ malignant neoplasias (PrePN 8.4 ± 0.7 years, PostPN 8.1 ± 0.3 years; Mann–Whitney U test = 31.0, *p* = 0.446). MCTs were diagnosed multiple times for some bitches throughout their available health history. There were sixteen MCT episodes in total (seven in PrePN bitches and nine in PostPN bitches).

**Figure 4:**
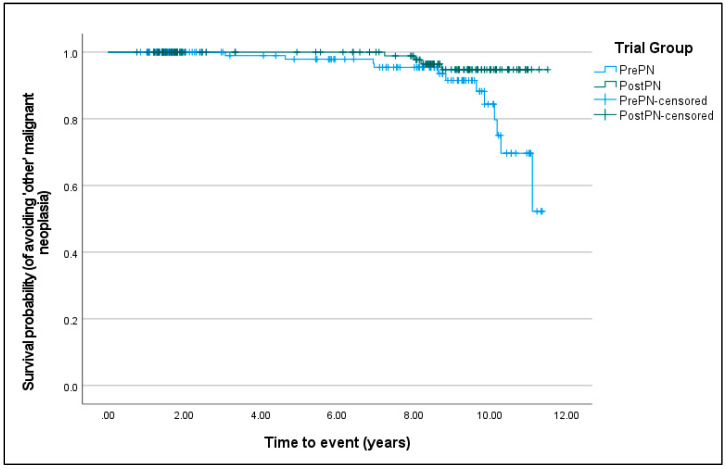
Kaplan–Meier curves of the survival probability (from having ‘other’ malignant neoplasias) for bitches neutered before puberty (PrePN, blue) or after puberty (PostPN, green). Censored values (+) indicate the time that bitches left the programme.

For having at least one malignant neoplasia (χ^2^ = 1.086, D.F. = 1, *p* = 0.297), Kaplan–Meier survival curves were not different between PrePN and PostPN bitches. For ‘other’ malignant neoplasias, PostPN bitches had a significantly improved chance of avoiding disease development (χ^2^ = 5.063, D.F. = 1, *p* = 0.024; Figure 4).

#### 3.2.3. Urogenital Diseases and Pseudopregnancy

Excluding pseudopregnancy, there were 54 bitches (25 PrePN and 29 PostPN) diagnosed with at least one urogenital disease (Chi-square = 0.498, D.F. = 1, *p* = 0.480). There was no difference in the number of bitches diagnosed with zero (130 PrePN and 122 PostPN), one (20 PrePN and 24 PostPN) or two or more (five PrePN and five PostPN) urogenital diseases (Chi-square = 0.565, D.F. = 2, *p* = 0.754). The most commonly reported problem was urinary tract infection (UTI)/cystitis (12 PrePN and nine PostPN [four bitches were diagnosed prior to neutering]; Chi-square = 0.380, D.F. = 1, *p* = 0.538), followed by perivulvar dermatitis (eight PrePN and five PostPN [four bitches were diagnosed prior to neutering]; Chi-square = 0.644, D.F. = 1, *p* = 0.422), vulval/abnormal discharge (five PrePN and eight PostPN [five bitches were diagnosed prior to neutering]; Chi-square = 0.378, D.F. = 1, *p* = 0.538), urinary incontinence (UI) (one PrePN and six PostPN; Yates’ Chi-square = 2.448, D.F. = 1, *p* = 0.118), recessed/inverted vulva (four PrePN and one PostPN [one bitch was diagnosed prior to neutering]; Yates’ Chi-square = 0.761, D.F. = 1, *p* = 0.383), and vulval/vaginal disorder (one PrePN and two PostPN [one bitch was diagnosed prior to neutering]; Yates’ Chi-square = 0.001, D.F. = 1, *p* = 0.975). USMI (one PrePN and one PostPN) and vaginitis (zero PrePN and three PostPN [all bitches were diagnosed prior to neutering]) occurred in too few bitches for statistical analysis. Pseudopregnancy was reported in no PrePN bitches and 23 PostPN bitches (analysis not possible); one of these was diagnosed post-neutering. No bitches were diagnosed with pyometra, struvite urolithiasis/urinary calculi, urinary/reproductive tract tumours, or urinary tract disorders. Only one bitch with perivulval dermatitis had a BCS in their lifetime that represented having obesity. One of the four PrePN bitches with a health code for recessed/inverted vulva was categorised as severely affected with recurrent perivulval dermatitis and was also diagnosed with atopic dermatitis. This bitch required vulvoplasty, which resulted in the resolution of the perivulval dermatitis.

The IRR for perivulval dermatitis was 1.78 (95% CI = 0.58–5.44), for UTI/cystitis was 1.39 (95% CI = 0.65–2.97), and for vulval/abnormal discharge was 0.70 (95% CI = 0.23–2.13).

**Figure 5:**
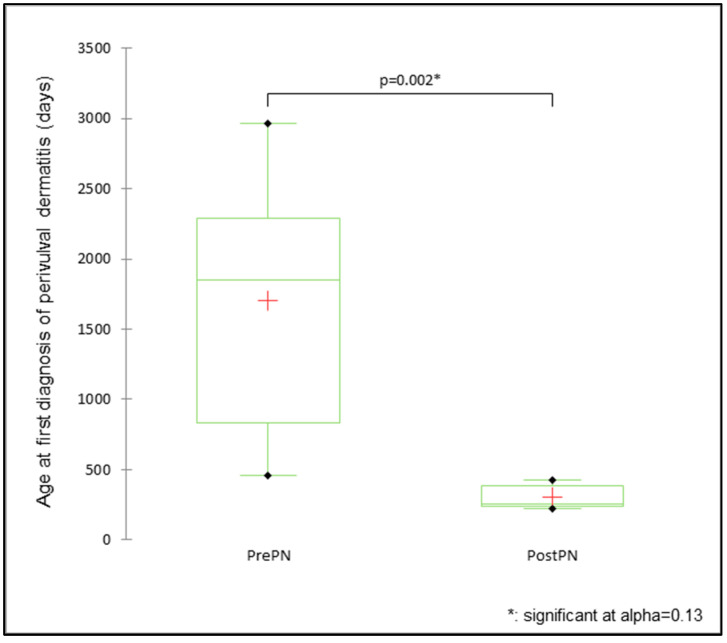
The mean, median, and range of days for the age at the diagnosis of perivulval dermatitis for bitches neutered prepubertally (PrePN, *n* = 8) or post-pubertally (PostPN, *n* = 5).

The mean age at the first diagnosis was not different between PrePN and PostPN bitches for UTI/cystitis (PrePN 1.9 ± 0.8 years, PostPN 2.9 ± 0.8 years; Mann–Whitney U test = 31.5, *p* = 0.115) or vulval/abnormal discharge (PrePN 2.6 ± 1.3 years, PostPN 1.7 ± 0.9 years; Mann–Whitney U test = 28.0, *p* = 0.264). There was a significant difference between PrePN and PostPN bitches in the mean age at the first diagnosis of perivulval dermatitis (Mann–Whitney U test = 40.0, *p* = 0.002), with the first diagnosis for PrePN bitches (4.7 ± 0.9 years) reported at significantly older ages than for PostPN bitches (0.8 ± 0.1 years; Figure 5). The effect size was 16.82. UI and UTI/cystitis were diagnosed multiple times for some bitches throughout their available health history. There were eight UI episodes in total (one in PrePN bitches and seven in PostPN bitches) and twenty-seven episodes of UTI/cystitis (15 in PrePN bitches and 12 in PostPN bitches).

For UTI/cystitis (χ^2^ = 0.610, D.F. = 1, *p* = 0.435) and having at least one urogenital disease (χ^2^ = 0.220, D.F. = 1, *p* = 0.639), Kaplan–Meier survival curves were not different between PrePN and PostPN bitches.

There were no significant associations between the vulval appearance and development data and urogenital diseases (Table 2).

#### 3.2.4. Immune Diseases

There were 138 bitches (80 PrePN and 58 PostPN) diagnosed with at least one immune disease (Chi-square = 5.384, D.F. = 1, *p* = 0.020). This was influenced by otitis and was no longer significant if otitis was excluded (at least one immune disease; 16 PrePN and 11 PostPN; Chi-square = 0.877, D.F. = 1, *p* = 0.349). There were no differences in the numbers of bitches diagnosed with zero (75 PrePN and 93 PostPN), one (69 PrePN and 50 PostPN) or two or more (11 PrePN and eight PostPN) immune diseases (Chi-square = 5.385, D.F. = 2, *p* = 0.068). The most commonly reported problem was otitis (74 PrePN and 55 PostPN [13 bitches were diagnosed prior to neutering]; Chi-square = 4.018, D.F. = 1, *p* = 0.045) followed by atopy (12 PrePN and nine PostPN; Chi-square = 0.380, D.F. = 1, *p* = 0.538). Autoimmune haemolytic anaemia (AIHA) (one PrePN and zero PostPN), hypothyroidism (one PrePN and zero PostPN), immune-mediated arthritis (two PrePN and zero PostPN), immune-mediated thrombocytopenia (one PrePN and zero PostPN), and inflammatory bowel disease (IBD) (zero PrePN and two PostPN) occurred in too few bitches for statistical analysis. No bitches were diagnosed with hypoadrenocorticism or systemic lupus erythematosus.

The IRR for atopy was 1.48 (95% CI = 0.63–3.52) and for otitis was 1.47 (95% CI = 1.15–1.88).

**Figure 6:**
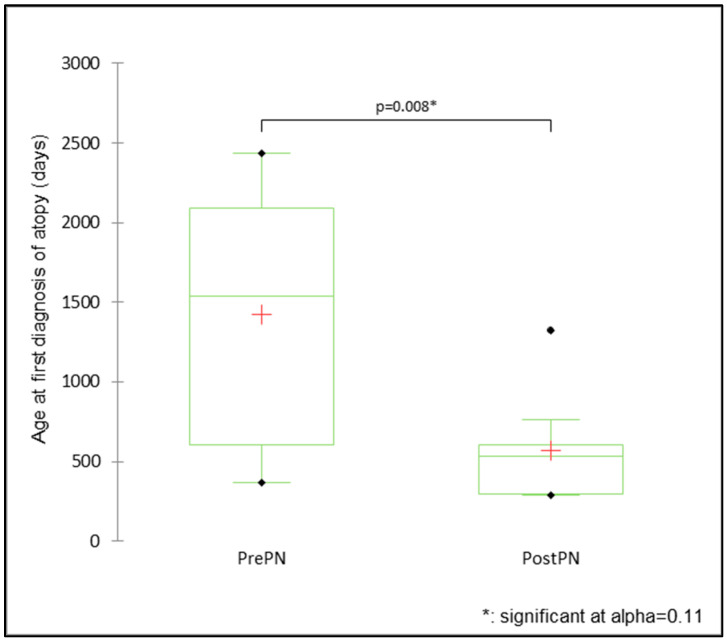
The mean, median, and range of days for the age at diagnosis of atopy for bitches neutered prepubertally (PrePN, *n* = 12) or post-pubertally (PostPN, *n* = 9).

The mean age at the first diagnosis was significantly different between PrePN and PostPN bitches for otitis (PrePN 1.7 ± 0.2 years, PostPN 2.1 ± 0.3 years; Mann–Whitney U test = 1676.5, *p* = 0.088). The effect size was −0.219, which was smaller than that determined by the power analysis (0.59). There was a significant difference in the mean age at the first diagnosis of atopy, with the first diagnosis for PrePN bitches (3.9 ± 0.6 years) reported at significantly older ages than for PostPN bitches (1.6 ± 0.3 years; Mann–Whitney U test = 90.5, *p* = 0.008; Figure 6). The effect size was 2.78. Otitis was diagnosed multiple times for some bitches throughout their available health history. There were 255 otitis episodes in total (145 in PrePN bitches and 110 in PostPN bitches).

**Figure 7:**
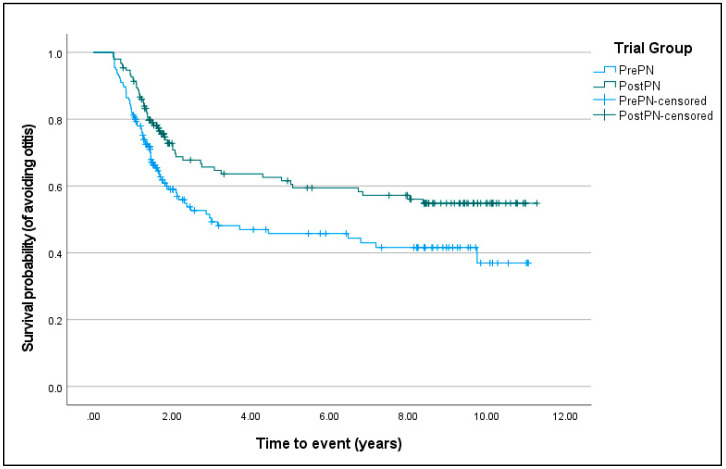
Kaplan–Meier curves of the survival probability (from having otitis) for bitches neutered before puberty (PrePN, blue) or after puberty (PostPN, green). Censored values (+) indicate the time that bitches left the programme.

For atopy, Kaplan–Meier survival curves were not different between PrePN and PostPN bitches (χ^2^ = 0.569, D.F. = 1, *p* = 0.451). For having at least one immune disease (χ^2^ = 8.413, D.F. = 1, *p* = 0.004) and otitis (χ^2^ = 7.090, D.F. = 1, *p* = 0.008; Figure 7), PostPN bitches had a significantly improved chance of avoiding disease development. The difference between curves for having at least one immune disease was influenced by otitis and was no longer significant if otitis was excluded (χ^2^ = 1.193, D.F. = 1, *p* = 0.275).

#### 3.2.5. Other Diseases

The most commonly reported problem was being overweight or having obesity (65 PrePN and 69 PostPN [seven bitches were diagnosed prior to neutering]; Chi-square = 0.439, D.F. = 1, *p* = 0.507). Histiocytoma was diagnosed in seven PrePN and three PostPN [one was diagnosed prior to neutering] bitches (Yates Chi-square = 0.851, D.F. = 1, *p* = 0.356). For all other diseases of interest, the numbers of bitches diagnosed were too small for statistical analysis: aortic stenosis (zero PrePN and two PostPN [one bitch was diagnosed prior to neutering]), diabetes mellitus (zero PrePN and one PostPN), early-onset cataract (two PrePN and two PostPN), and epilepsy (two PrePN and zero PostPN). No bitches were diagnosed with gastric volvulus or geriatric cognitive impairment. The IRR for being overweight or having obesity was 1.05 (95% CI = 0.75–1.47). The age at the first health record where a bitch was reported as being overweight or having obesity was not significantly different between PrePN (3.4 ± 0.3 years) and PostPN (2.9 ± 0.3 years) bitches (Mann–Whitney U test = 2562.5, *p* = 0.155). For being overweight and having obesity, Kaplan–Meier survival curves were not different between PrePN and PostPN bitches (χ^2^ = 0.143, D.F. = 1, *p* = 0.706).

One hundred seventy-nine bitches (90 PrePN and 89 PostPN) had an adult BCS recorded. There were 20 PrePN and 24 PostPN bitches that had an adult BCS of seven, eight, or nine and would therefore be considered as having obesity (Chi-square = 0.543, D.F. = 1, *p* = 0.461). The incidence rate ratio (IR) for having obesity based on the BCS was 0.93 (95% CI = 0.51–1.68).

### 3.3. Withdrawals from Working for Health Reasons

One hundred thirteen of the 195 bitches that qualified as assistance dogs had finished working as of 15 September 2023 (64 PrePN and 49 PostPN, Chi-square = 5.108, D.F. = 1, *p* = 0.024). Fifteen of these had been withdrawn from working for health reasons (11 PrePN and four PostPN). There was a significant difference in the number withdrawn for health reasons and those still working between PrePN and PostPN bitches (Chi-square = 5.602, D.F. = 1. *p* = 0.018). Bitches were withdrawn for health reasons between 2.4 and 8.9 years of age (PrePN bitches 6.0 ± 0.7 years, PostPN bitches 6.5 ± 1.1 years; Table 3). The most common reasons for health withdrawals from working were musculoskeletal diseases (six PrePN and two PostPN) and malignant neoplasias (two PrePN and one PostPN). Two PrePN bitches were withdrawn for osteoarthritis and two for CCL rupture. The final four health withdrawals from working were for USMI (PostPN), autoimmune thrombocytopenia (PrePN), idiopathic generalised epilepsy (PrePN), and bilateral cataracts (PrePN).

### 3.4. Cause of Death

As of 15 September 2023, 32 bitches had died (18 PrePN and 14 PostPN; Chi-square = 0.448, D.F. = 1, *p* = 0.503). The age at death was between 3.2 and 10.8 years of age (PrePN mean = 7.9 ± 0.6 years, PostPN mean = 8.6 ± 0.4 years; Mann–Whitney U test = 118, *p* = 0.779; Table 3).

The most common cause of death was malignant neoplasia (13 PrePN and nine PostPN). Not all bitches were subject to a post-mortem examination; therefore, histology to confirm type of neoplasia was not always available and the body system affected was described where a histological examination was not performed (Table 4). Other causes of death were autoimmune haemolytic anaemia (one PrePN), seizures (one PostPN), renal disease (one PostPN), severe peritonitis (one PrePN), and one PrePN bitch was euthanised due to osteoarthritis. Exact causes of death were unknown for five bitches (two PrePN and three PostPN).

## 4. Discussion

In clinical practice, it is common for recommendations to be made regarding neutering timing for bitches relative to the occurrence of puberty or the first oestrous. Despite the large amount of literature relating to the impacts of neutering and the timing of neutering relative to age on bitch health, the effect of neutering before or after puberty has not been well studied [10]. This study followed a cohort of bitches to examine the impact of neutering before or after puberty on the occurrence of several diseases throughout life. Detrimental associations between prepubertal neutering and musculoskeletal diseases were identified that may be expected, based on the previous literature relating to the impact of neutering at different ages. There were interesting differences for immune diseases that may be attributable to pubertal status at neutering, such as an older age at the diagnosis of atopy and lower probability of remaining free from otitis externa for bitches neutered prepubertally, that have not been reported previously. Unexpectedly, no associations were identified between the pubertal status at neutering and the incidence of any urogenital diseases, with low numbers of bitches diagnosed with diseases such as UI, which is commonly associated with neutering. However, for many diseases, the number of bitches diagnosed was small and did not allow for statistical comparisons. Significantly more pre- than post-pubertally neutered bitches had finished working, and more had been withdrawn from working for health reasons at the time of data collation. For diseases diagnosed in enough bitches to enable an analysis, the results suggest that prepubertal neutering may have a detrimental effect on some future musculoskeletal diseases, immune diseases, and on working life in female dogs of the crossbreeds studied. This provides important information to support neutering policies and to help maintain optimal dog welfare.

The results of the study are limited by the characteristics of the study sample; only female Labrador and Golden Retriever crossbreeds were included from one assistance dog programme in the UK. This may affect the applicability of findings to other breeds of dog and dogs with different genetic predisposition to disease. The limited number of bitches available to recruit for the study resulted in some of the less common diseases being diagnosed in too few bitches to enable comparisons between groups, or to include in more complex models to examine the effect of trial group while accounting for the effects of covariates. These were diseases commonly associated with neutering in the literature, such as HD (three bitches diagnosed), ED (six bitches diagnosed), HSA (three bitches diagnosed), and LSA (four bitches diagnosed). While this limited the data analysis that was possible, it also raised important considerations for other authors. There is a need for controlled prospective studies of well-managed populations with known histories and similar environmental experience. However, there are benefits to the present study design, wherein a prospective study was conducted in a carefully controlled population, which minimised some of the risks that can be associated with retrospective studies and those conducted on larger numbers of animals but with varied or unknown backgrounds.

One of the major limitations was that health data were only available for bitches until the time they left the assistance dog programme. More PrePN than PostPN bitches had ceased working at the time of health data collation, which could affect the results due to data not being available for the bitches once they were rehomed. Attempts to address this by using Cox regression analysis to deal with censored data were affected by the small number of bitches in the study. Even with the calculated disease rate per 1000 dog-days used to standardise the presentation of disease incidence data, this limits the findings related to diseases that have a later age of onset. This limitation applies to both trial groups. There was no difference between groups in the number of PrePN and PostPN bitches within each group for years of health data available from six months of age to the last health record; therefore, the differences highlighted are valid. Some diseases were diagnosed prior to neutering; however, an analysis of data for the age at diagnosis would not be expected to highlight significant differences between groups if neutering timing was not associated with disease occurrence. In the present study, only malignant neoplasias were considered; future work could be expanded to include benign conditions of all body systems.

The results do provide an indication of the sample sizes that would be required in future prospective studies to adequately study these diseases, as well as information about the expected age of onset. For instance, for an analysis of some disease data, over 2000 animals would have been required. Studying much larger populations of dogs would provide more confidence in the results and allow for analyses of the diseases of interest, such as ED and HD, that were not possible in the present study. However, such a large study may not be feasible in a controlled population; the present study utilised dogs from one of the world’s largest assistance dog training programmes, and the recruitment of large numbers of dogs comparable to those in retrospective studies of veterinary databases would not have been possible. Using dog data from existing data sources, such as large veterinary databases, would allow the recruitment of the sample sizes required. However, issues such as the retrospective examination of data, incomplete data, recall bias, limited information about potential confounding and environmental factors, and inconsistent dog rearing and husbandry, would then impact the quality of the results. Within the present study, the well-managed and monitored population with controlled data recording reduced the likelihood of missed diagnoses.

In a recent scoping review [10], no previous studies were identified that examined the impact of neutering in relation to puberty on musculoskeletal diseases. The results of the present study suggest that neutering bitches prepubertally may be negatively associated with musculoskeletal health outcomes, consistent with the results reported by others related to the neutering age [1,8,9,22,23,24]. There were significantly more PrePN bitches with CCL rupture, although the small number of PostPN bitches affected may have affected the test reliability, and the incidence rate ratio was large (17.8). A greater risk of CCL disease is frequently associated with earlier neutering [1,22,23]. Ekenstedt et al. [23] reported that 87% of the CCL rupture cases for bitches in their case–control study were neutered at or before 12 months of age. Torres de la Riva et al. [1] reported no CCL disease in Golden Retriever bitches neutered at or after 12 months of age compared to 7.7% for bitches neutered at younger than 12 months of age, with a mean age of onset of 4.8 years. The mean age at the first diagnosis in the present study for PrePN bitches was 7.1 years.

Associations between prepubertal neutering and musculoskeletal diseases may be mediated in part by gonadal hormones, or the earlier absence thereof, leading to high circulating luteinising hormone (LH) concentrations, as proposed for CCL disease [25]. LH receptors have been identified in the femoral head and hyaline cartilage, although differences in expression between entire and neutered dogs have not been demonstrated [26]. Reproductive hormones have been shown to affect CCL laxity in humans [27,28,29]. Reproductive hormones have also been shown to affect collagen concentrations and ligament strength in rabbit cruciate ligaments [30,31]. Kutzler [25,26] proposes that the continuous activation of LH receptors (caused by persistently elevated LH concetrations) in the cruciate ligament could increase ligament laxity, leading to joint instability.

There was a significant difference between the survival curves both for osteoarthritis and for having at least one musculoskeletal disease. The probabilities of remaining free from disease for PrePN bitches for both conditions decreased at earlier ages than for PostPN bitches. Osteoarthritis was diagnosed in PrePN bitches at significantly younger ages than in PostPN bitches, although the effect size was small. While more PrePN bitches were diagnosed with osteoarthritis and at least one musculoskeletal disease than PostPN bitches, the differences in incidence between groups were not significant. There have been a limited number of studies related to the impacts of the neutering age on osteoarthritis, with most authors proposing that the differences in other diseases, such as HD and ED, may predispose dogs to osteoarthritis later in life. Spain et al. [9] investigated the effect of the age at neutering on numerous medical conditions and found no association between the age at neutering and arthritis. In comparison to the study by Spain et al. [9], it is possible that the bitches in the present study had more regular health checks and were more likely to be presented to veterinarians for an investigation of possible arthritis due to the potential impact on their working ability. The osteoarthritis observed in the present study may not have been associated with HD or ED, with only small numbers of bitches being diagnosed (1% of bitches overall were diagnosed with HD). Hart et al. [4] reported HD in between 2.1 and 9.6% of Golden Retriever and Labrador bitches. HD and ED are complex diseases that are influenced by many factors. The low proportions observed could be influenced by selective breeding and good environmental management and husbandry practices for the bitches in the programme.

Along with musculoskeletal disease, the development of neoplasia is a commonly investigated outcome, although only one study was identified that considered the pubertal status at the time of neutering [32]. Scheider et al. [32] reported an increased relative risk of malignant mammary neoplasia in bitches neutered after puberty compared to bitches neutered before puberty. Other studies that have compared bitches neutered at different ages reported similar effects on mammary neoplasia [1,33]. Mammary neoplasia was not observed in any bitch in the present study, although only 68 bitches had health data to at least 9.5 years of age; the remaining bitches were either still too young or had been withdrawn and rehomed at younger ages. The mean age at onset of malignant mammary neoplasia in the bitch has been reported as between 9.5 to 14 years [34,35,36,37,38], and is most commonly observed in entire bitches and those neutered after experiencing several oestrous cycles [32,39]. It may be useful to re-examine data for mammary neoplasia once lifetime information is available, though with the low expected incidence and considering the number of bitches in the present study, statistical comparisons may not be possible.

Other types of neoplasias, such as appendicular bone sarcoma [40], HSA, and MCTs [1], have been associated with the age at neutering. However, these associations are not similar across different types of neoplasias, with an earlier neutering age associated with a decreased risk of some and an increased risk of others. In 242 Golden Retriever bitches, Torres de la Riva et al. [1] reported that those neutered before 12 months of age were significantly less likely to have HSA and MCTs compared to bitches neutered at 12 months of age or older. While that study did not consider the pubertal status at the time of neutering, the results of the present study were similar for MCTs, with more PostPN bitches being diagnosed, although the numbers of bitches were small (four PrePN and seven PostPN) and the difference was not significant. In contrast, for LSA, Torres de la Riva et al. [1] reported that, although not significant, a higher percentage of bitches in the early (5.9%) rather than late neutering group (1.4%) was diagnosed. In the present study, LSA was diagnosed in too few bitches (1.4% in both groups) for analysis, with an incidence similar to that for late neutered bitches reported by Torres de la Riva et al. [1]. Cooley et al. [40] also found an increased risk with a younger neutering age when investigating bone sarcoma in a study that included 389 female Rottweilers. In the present study, no bitches were diagnosed with osteosarcoma.

There were no significant differences in the number of PrePN compared to PostPN bitches diagnosed with any of the individual types of malignant neoplasias studied, and most were diagnosed in too few bitches for statistical analysis. However, for ‘other’ malignant neoplasias, numerically more PrePN than PostPN bitches were diagnosed (7.7% PrePN and 2.6% PostPN) and the incidence rate ratio was 3.62 with a 95% CI of 1.18 to 11.09. There was also a significant difference in survival curves with the probability of remaining free from ‘other’ malignant neoplasias for PrePN bitches decreasing to around 0.8 by just after 10 years of age and continuing to decline thereafter compared to that for PostPN bitches, which appeared to plateau at ~0.95 from around 9.5 years of age. Increased incidences of neoplasias have been well documented in neutered compared to entire dogs, with higher circulating LH concentrations considered a potential contributing factor. LH receptors have been identified in smooth muscle and vascular endothelial cells, lymphoid tissues and lymphocytes, and the skin, and in HSAs, MCTs, and cells from neoplastic lymph nodes [25,26,41]. The potential effects of high circulating LH concentrations and activation of these LH receptors and the mechanisms by which disease states are influenced are largely unknown. For the present study, the findings related to malignant neoplasias may be influenced by the small number of bitches diagnosed, and it will be useful to re-examine the data once more bitches have reached older ages. The low incidence combined with the late age of onset means that these diseases may have less impact on the dog’s working life. However, the impacts for dog and owner wellbeing should still be considered.

In the present study, the number of bitches diagnosed with urogenital diseases was low across all diseases, and there were no significant differences in the number of PrePN compared to PostPN bitches diagnosed with any urogenital disease. For some diseases, such as pyometra and reproductive tract tumours, the fact that all bitches were neutered either prepubertally or within 195 days of their first season and that ovariohysterectomies were performed would largely exclude the risk of disease. The lack of apparent associations between other urogenital diseases and the pubertal status at neutering, and between markers of vulval development and future disease was unexpected. Urogenital diseases, especially UI, are perhaps one of the most frequently investigated factors when considering the timing of neutering for female dogs, specifically the appropriate development of the external genitalia and proposed related impacts on future disease. While neutering at any age is suggested to increase the risk of UI [42], the present study only considered the relationship to the pubertal status at neutering. Some authors suggest that the removal of oestrogen at an early age may impact normal vulval development and that this can predispose animals to urogenital diseases [15,43,44]. Verstegen-Onclin and Verstegen [45] reported high proportions of bitches neutered before puberty in a population of bitches in Florida presented to a specialist small animal reproduction clinic for urogenital problems, including recessed vulva, vaginitis, vaginal dermatitis, and UTI. The authors suggest that these problems may occur at a higher incidence in early neutered bitches. However, it is possible that this method of presenting their data may be open to misinterpretation. It may be correct that for bitches with disease, high numbers were neutered at earlier ages. However, in a prospective study design—the most appropriate study design for examining risk factors—where bitches are followed throughout life to examine occurrence of disease, the incidence may be lower than that which may be expected from solely studying data for dogs with the disease.

A recent scoping review [10] identified four studies that categorised bitches as pre- or post-pubertal at surgical neutering and investigated outcomes related to incontinence. Two of the studies reported no significant effect [46,47], while two did report an effect of neutering before or after puberty, but with conflicting results. Stocklin-Gautschi et al. [48] suggested that the incidence of UI in bitches neutered prepubertally was lower compared to those neutered post-pubertally using data from two different study populations. Lutz et al. [49] reported the opposite, with significantly more bitches neutered prepubertally being reported with UI than those neutered post-pubertally. The method of grouping bitches or data irregularities may, however, have compromised the findings reported by Lutz et al. [49]; the prepubertally neutered group included some bitches aged up to 1.4 years, and some ‘post-pubertally neutered’ bitches were allegedly aged 0.3 years. Both increased [9,42,50] and decreased [51] risks of UI have been reported for bitches neutered at earlier ages. Other studies have reported no association between the incidence of UI and bitch neutering age [6,52,53]. More recently, using a target trial emulation approach for 612 bitches neutered between three and seven months of age and 888 bitches of various breeds neutered between seven and 18 months of age, Pegram et al. [54] reported a reduced risk of UI for bitches neutered at later ages (OR 0.80, 95% CI 0.54 to 0.97). In the present study, UI occurred in low numbers (2.3% of bitches overall), and more frequently in bitches neutered after puberty (one PrePN and six PostPN). In other studies, rates of UI incidence have been reported from 0 to 20% for neutered bitches [4,5,42,55], although the incidence is known to vary by breed [55,56]. The overall incidence reported in the present study is similar to reports for neutered purebred Golden Retrievers and Labradors by others. Hart et al. [4] reported no cases in Golden Retrievers neutered at any age and 2–3% UI in Labradors neutered up to 12 months of age, and Pegram et al. [42] reported an incidence of 2.9% for Labradors.

While there were no significant differences in the urogenital disease incidence in the present study, some differences were identified between PrePN and PostPN bitches for the age at disease diagnosis. The age at the first diagnosis of perivulval dermatitis was older for PrePN bitches, and most PostPN bitches affected with perivulval dermatitis were diagnosed before neutering (four out of five bitches). The reason for this difference in age is unclear, although the finding for the age at the diagnosis of perivulval dermatitis is similar to that observed for atopy. Thus, there is the potential for the same causal factor to be affecting the skin around the vulva as well as the skin elsewhere, although only two of the bitches with perivulval dermatitis were also diagnosed with atopy.

The impact of neutering timing on immune diseases has historically received less focus than other health outcomes, yet in the present study, there were some interesting findings. Otitis externa was the most commonly reported immune disease and, while there were not significantly more prepubertally neutered bitches diagnosed with otitis (at an alpha of 0.04; 74 PrePN and 55 PostPN), there were significant differences in survival curves. The probabilities of remaining free from otitis for PrePN bitches by two years of age were approximately 0.6 compared to over 0.7 in PostPN bitches, and by four years of age were approximately 0.45 for PrePN bitches compared to over 0.6 for PostPN bitches. The age at the diagnosis of atopy was significantly older for PrePN than PostPN bitches and potentially older than may be expected. Signs of atopy are usually observed in this assistance dog population by 12 to 18 months of age, and, following a period of clinical work-up, a diagnosis is normally confirmed within four to six months (Adams, personal communication [57]). Besides atopy and otitis, other immune diseases were diagnosed in too few bitches for further statistical analysis, including hypothyroidism (one PrePN and zero PostPN), which is suggested by some authors to be more common in neutered bitches due to the effect of neutering on thyroid function [58,59].

Atopy is a complex disease influenced by many environmental and genetic factors. It is understood that sex hormones influence immune responses and skin permeability in rats [60]. Sundburg et al. [61] reported an increased risk of atopy in neutered compared to entire female dogs and suggested that oestrogens may have a suppressive effect on disease occurrence. However, that would not explain the difference in the age at diagnosis reported here, nor would the longer period exposed to unregulated and high LH concentrations explain why bitches neutered earlier were diagnosed at older ages. There may be an effect of experiencing puberty: in humans, before puberty, atopic dermatitis is more common in males than females, while the reverse is true after puberty [60]. Bitches in the present study that experienced puberty may have experienced earlier triggers for atopy than those that did not. Serial measurements of circulating gonadal hormones would have been useful to understand associations between these factors and the development of atopy; however, they were not undertaken in the present study. Further investigation into the roles of gonadal hormones and skin function in the dog and larger studies involving dogs neutered at different times would be useful to increase understanding of the potential effects of gonadal hormones on canine atopic dermatitis.

For diseases in the ‘other’ category, there were very few bitches diagnosed with any condition except for being overweight or having obesity. It is generally accepted that neutering at any age increases the risk of being overweight [62,63], and some work has suggested associations between the neutering age and the likelihood of dogs becoming overweight [8,9]. However, in the present study considering the pubertal status at neutering, there was no difference between PrePN and PostPN bitches in the number that were overweight or had obesity, in the age at the first report of being overweight or having obesity, or between the survival curves. This may be due to the small relative difference in neutering age between the PrePN and PostPN groups. Interestingly, although the differences were not significant, fewer PrePN than PostPN bitches were found to be overweight or have obesity (41.9% vs. 45.7%) or have a BCS indicating obesity (12.9% vs. 15.9%). This is similar to the findings of Spain et al. [9], who reported a decreasing risk of overweight body condition with decreasing age at neutering for 1659 dogs of various breeds, although results were not reported separately for male and female dogs. Simpson et al. [8] examined data for over 2700 Golden Retrievers and found the opposite relationship, reporting that dogs neutered at 6–12 months had an increased risk of overweight/obesity compared to those neutered at >12 months of age, with no difference in the risk for dogs neutered at <6 months of age. There were differences in the study design, with all dogs in the study by Spain et al. [9] being neutered between six weeks and 12 months of age. It would be interesting to examine data from the present study in more detail to determine whether bitches were only classed as overweight for a short period or whether being overweight was a longer-term problem. It would also be interesting to examine whether associations that would be expected between diseases such as being overweight and forelimb lameness were present, and to examine other potential associations such as otitis and being overweight. However, these were not within the scope of the study.

The impact of neutering timing in relation to puberty on future health is of interest for all dogs; however, for assistance dogs, where there may be an effect on the ability of the dog to continue working with their human partner, it is particularly important. In the present study, significantly more prepubertally neutered bitches had finished working at the time of data collation. Additionally, significantly more had been withdrawn from working for health reasons, including CCL rupture and osteoarthritis, the latter occurring at significantly younger ages in PrePN bitches. Differences in the number of bitches that had died and age at death between the two groups were not significant; however, at the time of data collation, not all bitches had reached the end of life. It will be interesting to examine these variables again once whole-life data are available, and to see whether the difference in the age at death becomes significant once data are available for all bitches.

The differences observed may be related to gonadal hormones and levels of exposure. Bitches neutered after puberty have experienced elevated oestrogen and concentrations and then elevated LH concentrations for less time. It is possible that exposure to oestrogen and progesterone and then unregulated and high LH concentrations have impacts. Cruciate disease, neoplasia, and skin disorders such as otitis and atopy may be influenced by a component of oestrogen and progesterone in terms of the bone or skin itself, as well as the potential influence on receptors. Any differences observed would be small in comparison to studies such as those by Hart et al. [2,3], which examined data for bitches neutered over a greater age range (less than six months to eight years) with an associated greater difference in high LH exposure, and are likely linked to whether the bitches have experienced an increase in oestrogen or progesterone concentrations (experienced puberty) or not. Concentrations of these reproductive hormones were not measured in this study’s bitches, and this could be recommended for future studies.

Alongside a greater understanding of the actions of LH and FSH in disease development, further long-term investigations are required to understand the risks and benefits of the long-term use of GnRH analogues as a medical strategy to potentially replace or delay neutering. Studies should be conducted that control for or limit confounding variables and that confirm the pubertal status [64]. It is possible that, after some years of living with repeated deslorelin implants commencing prior to puberty (to prevent the induction of oestrus observed after implantation in post-pubertal anoestrus bitches), bitches could be neutered later and therefore have fewer years with high and unregulated LH levels. Alternatively, GnRH analogues implanted following ovariohysterectomy or ovariectomy could be used to maintain low gonadotrophin concentrations. These methods could reduce future risks of developing diseases that may be associated with LH, but prospective cohort studies with large numbers of bitches, or potentially studies involving rodent models, are required to further investigate this [25,64]. Additionally, future studies could consider alternative methods to prevent reproduction in dogs. Surgical neutering via hysterectomy, therefore removing the potential for unplanned pregnancies and uterine disease while leaving the ovaries in place and not disrupting the hypothalamus–anterior pituitary–gonad axis may be a useful alternative to surgeries that remove the ovaries. However, there are limited studies available considering the long-term outcomes of hysterectomy for bitches, and there are considerations for owners; behaviours influenced by gonadal hormones would persist and there would be no prevention of ovarian disease or other diseases associated with ovarian hormones.

Overall, considering the disease incidence and age at diagnosis, there were negative associations between prepubertal neutering and musculoskeletal diseases and immune diseases, as well as for ‘other’ malignant neoplasias. Furthermore, there were potential detrimental consequences for the working life. These findings, combined with recently published recommendations for neutering age [4,5], would suggest that if bitches of these breeds are to be neutered, doing so after known puberty may be beneficial from a health perspective based on the diseases investigated.

## 5. Conclusions

The results of the present study suggest that for Labrador/Golden Retriever crossbreed bitches, there may be detrimental associations between neutering before known puberty and musculoskeletal diseases, particularly for CCL disease, and immune diseases, specifically otitis. Additionally, there may be associations with the age at diagnosis for diseases that could affect the working ability and health management of assistance dogs, such as a delayed onset of atopy and earlier onset of osteoarthritis. In contrast, there was little to no association with the individual types of malignant neoplasias studied or the incidence of urogenital diseases. However, there are important limitations to consider with regard to the number of bitches in the study, the limited breeds used, the small number of bitches diagnosed with diseases, and the fact that lifetime data were not evaluated for all bitches. The study provides important information for other researchers in the field in terms of the size of study populations needed for valid statistical analyses.

These findings will be of interest to assistance and working dog organisations, veterinarians, and pet dog owners, as they provide additional information to improve understanding of the future health impacts of neutering bitches at specific time points in relation to known puberty.

## Figures and Tables

**Table 1:** The number and breed of bitches in this study investigating the effect of neutering before (PrePN) or after (PostPN) puberty. The sire breed is shown first.

Breed	PrePN Bitches	PostPN Bitches
Golden Retriever cross Labrador	100	104
Golden Retriever cross (Labrador cross Golden Retriever)	0	1
Labrador cross Golden Retriever	24	19
Labrador cross (Golden Retriever cross Labrador)	28	22
Labrador cross (Labrador cross Golden Retriever)	3	5
Total	155	151

**Table 2:** The percentage of bitches with and without a diagnosis of each urogenital disease that also had each type of vulval appearance and percentage dorsal fold coverage at 17 months of age and each descriptor for vulval growth between six and 17 months of age. Results from Chi-square analyses are shown. An analysis was not possible for dorsal fold coverage for urinary incontinence due to the small numbers of affected bitches with vulval growth data available.

Disease	Bitches Diagnosed with the Disease	Bitches Unaffected by the Disease	Chi-Square Analysis
Perivulval dermatitis (*n* = 13 diagnosed)			
Vulval appearance	Normal = 53.8%	Normal = 53.2%	Chi-square = 0.129, *p* = 0.720
	Recessed = 46.2%	Recessed = 30.0%	
	Juvenile = 0	Juvenile = 3.1%	
	Recessed and juvenile = 0	Recessed and juvenile = 4.1%	
	No image = 0	No image = 9.6%	
Dorsal fold coverage (%)	≤20% = 15.4%	≤20% = 24.2%	Yates Chi-square = 0.216, *p* = 0.642
	30% = 23.1%	30% = 17.7%	
	40% = 7.7%	40% = 8.9%	
	50% = 7.7%	50% = 3.1%	
	No image = 46.2%	No image = 46.1%	
Vulval growth	Same = 7.7%	Same = 11.6%	Yates Chi-square = 0.001, *p* = 0.982
	Smaller = 30.8%	Smaller = 28.3%	
	Larger = 38.5%	Larger = 48.5%	
	Not measured = 23.1%	Not measured = 11.6%	
Urinary incontinence (*n* = 7 diagnosed)			
Vulval appearance	Normal = 71.4%	Normal = 52.8%	Yates Chi-square = 0.095, *p* = 0.758
	Recessed = 14.3%	Recessed = 31.1%	
	Juvenile = 0	Juvenile = 3.0%	
	Recessed and juvenile = 14.3%	Recessed and juvenile = 3.7%	
	No image = 0	No image = 9.4%	
Dorsal fold coverage (%)	≤20% = 0	≤20% = 24.4%	N/A
	30% = 28.6%	30% = 17.7%	
	40% = 0	40% = 9.0%	
	50% = 0	50% = 3.3%	
	No image = 71.4%	No image = 45.5%	
Vulval growth	Same = 14.3%	Same = 11.4%	Yates Chi-square = 1.660, *p* = 0.198
	Smaller = 0	Smaller = 29.1%	
	Larger = 85.7%	Larger = 47.2%	
	Not measured = 0	Not measured = 12.4%	
UTI/cystitis (*n* = 21 diagnosed)			
Vulval appearance	Normal = 42.9%	Normal = 54.0%	Chi-square = 1.651, *p* = 0.199
	Recessed = 42.9%	Recessed = 29.8%	
	Juvenile = 4.8%	Juvenile = 2.8%	
	Recessed and juvenile = 4.8%	Recessed and juvenile = 3.9%	
	No image = 4.8%	No image = 9.5%	
Dorsal fold coverage (%)	≤20% = 19.0%	≤20% = 24.2%	Yates Chi-square = 0.053, *p* = 0.818
	30% = 19.0%	30% = 17.9%	
	40% = 4.8%	40% = 9.1%	
	50% = 9.5%	50% = 2.8%	
	No image = 47.6%	No image = 46.0%	
Vulval growth	Same = 23.8%	Same = 10.5%	Chi-square = 0.437, *p* = 0.509
	Smaller = 23.8%	Smaller = 28.8%	
	Larger = 42.9%	Larger = 48.4%	
	Not measured = 9.5%	Not measured = 12.3%	
Vulval/abnormal discharge (*n* = 13 diagnosed)			
Vulval appearance	Normal = 53.8%	Normal = 53.2%	Chi-square = 0.129, *p* = 0.720
	Recessed = 38.5%	Recessed = 30.4%	
	Juvenile = 0	Juvenile = 3.8%	
	Recessed and juvenile = 7.7%	Recessed and juvenile = 3.1%	
	No image = 0	No image = 9.6%	
Dorsal fold coverage (%)	≤20% = 38.5%	≤20% = 23.2%	Yates Chi-square = 0.002, *p* = 0.960
	30% = 30.8%	30% = 17.4%	
	40% = 7.7%	40% = 8.9%	
	50% = 0	50% = 3.4%	
	No image = 23.1%	No image = 47.7%	
Vulval growth	Same = 0	Same = 11.9%	Chi-square = 0.732, *p* = 0.392
	Smaller = 30.8%	Smaller = 28.3%	
	Larger = 61.5%	Larger = 47.4%	
	Not measured = 7.7%	Not measured = 12.3%	

*n* = number of bitches.

**Table 3:** The minimum, maximum, and mean (± SEM) age at withdrawal from working as an assistance dog for health reasons for 15 bitches, and age at death for 32 bitches that were neutered before (PrePN) or after (PostPN) puberty.

Descriptive Statistic	PrePN	PostPN
Age at withdrawal from working for health reasons		
Number of bitches	11	4
Mean (SEM) (years)	6.2 (0.7)	6.5 (1.1)
Range (years)	2.4–8.9	3.3–8.3
Age at death		
Number of bitches	18	14
Mean (SEM) (years)	7.9 (0.6)	8.6 (0.4)
Range (years)	3.2–10.3	5.2–10.8

**Table 4:** The type of neoplasia noted as cause of death for 22 bitches neutered before (PrePN) or after (PostPN) puberty.

Neoplasia Noted/Primary Organ Affected	PrePN (N)	PostPN (N)
Haemangiosarcoma	1	2
Intestinal	0	1
Liver	1	1
Lung	2	1
Lymphoma	2	2
Mast cell tumour	1	1
Neuroendocrine carcinoma	1	0
Soft tissue sarcoma	1	0
Spinal cord	0	1
Spleen	2	0
Stomach	1	0
Not reported	1	0

## Data Availability

Restrictions apply to the availability of these data. Data were obtained from Guide Dogs UK and are available from Rachel Moxon with the permission of Guide Dogs.

## Supplementary Materials

The following supporting information can be downloaded at https://www.mdpi.com/article/10.3390/ani15020167/s1: Supplementary Material S1. Diseases chosen for inclusion in the study; Supplementary Material S2. The health codes searched for each disease; Supplementary Material S3. G*Power-determined values of alpha for each analysis; and Supplementary Material S4. Descriptive data for bitches diagnosed with each disease.
