# Supplementary material for: A Prospective Study Investigating the Health Outcomes of Bitches Neutered Prepubertally or Post-Pubertally
Source: Animals (Basel). 2025 Jan 10;15(2):167. doi: 10.3390/ani15020167 (PMC11758339; doi:10.3390/ani15020167)
Supplement: Supplementary file 1 [file animals-15-00167-s001.zip › Animals Supplementary Materials 1 Diseases chosen for inclusion.pdf]

Supplementary materials 1 - The diseases that were chosen for inclusion in the study and the reason for inclusion.

Health condition	Health reasons for withdrawal	Guide Dogs Chief veterinary Officer / Breeding Dog Health Specialist	Survey of veterinarians	Scientific literature	Veterinary student projects
**Musculoskeletal (joint/orthopaedic)**					
Cruciate disease	1			1	
Elbow dysplasia	1				
Forelimb lameness		1	1		
Hip dysplasia				1	1
Juvenile osteochondrosis					
Osteochondritis dissecans		1			
Osteoarthritis				1	
Patella luxation				1	
**Malignant neoplasia**					
Adenoma / adenocarcinoma			1	1	
Fibroma/fibrosarcoma			1		1
Haemangiosarcoma				1	
Lymphosarcoma/lymphoma				1	
Mammary neoplasia				1	
Mast cell tumour				1	
Melanocytic tumour				1	
Osteosarcoma				1	
Squamous cell carcinoma				1	
Transitional cell carcinoma				1	
Urogenital					
Urinary / reproductive tract tumour				1	
Perivulvar dermatitis			1	1	1

Pseudopregnancy			1	1	1
Pyometra			1	1	1
Recessed/inverted/juvenile vulva					1
Struvite urolithiasis / urinary calculi				1	
Urinary incontinence			1		1
Urinary tract disorders					1
Urinary sphincter mechanism incompetence			1		1
Urinary tract infection / cystitis				1	1
Vaginal / vulval disorder				1	1
Vaginitis			1	1	
Vulval discharge, abnormal discharge			1		1
Immune					
Atopy	1	1	1		
Autoimmune haemolytic anaemia				1	
Hypoadrenocorticism				1	
Hypothyroidism				1	
Immune-mediated arthritis					
Immune-mediated thrombocytopenia				1	
Inflammatory bowel disease (IBD)				1	
Otitis externa		1			
Systemic lupus erythematosus					1
Other					
Aortic stenosis				1	
Bodyweight (obesity)		1	1		
Diabetes mellitus				1	
Early onset cataracts (eyes)				1	
Epilepsy (idiopathic) (CNS)				1	
Gastric volvulus (GI)				1	
Geriatric cognitive impairment / cognitive dysfunction syndrome				1	
Histiocytoma				1	
