# Supplementary material for: A Prospective Study Investigating the Health Outcomes of Bitches Neutered Prepubertally or Post-Pubertally
Source: Animals (Basel). 2025 Jan 10;15(2):167. doi: 10.3390/ani15020167 (PMC11758339; doi:10.3390/ani15020167)
Supplement: Supplementary file 1 [file animals-15-00167-s001.zip › Animals Supplementary Materials 2 The health code searches run for each health condition.pdf]

Supplementary materials 2 - The health code searches run for each health condition.
Search for dogs born 01 02 2012 to 31 08 2015

MUSCULOSKELETAL

Juvenile osteochondrosis
Juvenile osteochondrosis

Cruciate disease
Cruciate Ligament Disease
Rupture of cruciate ligaments

Elbow dysplasia
Canine elbow dysplasia
Short radius syndrome
Medial compartment disease
Short ulna syndrome
Fragmented coronoid process
Medial coronoid process disease
Ununited anconeal process
Canine trochlear notch incongruity
OCD - osteochondritis dissecans of the elbow

Forelimb lameness
Forelimb lameness
Forelimb stiffness

Hip dysplasia
Hip dysplasia
Unilateral dysplastic hip
Bilateral dysplastic hip
Subluxation of hip joint

Osteoarthritis
Arthritis (check for osteoarthritis)
Chronic osteoarthritis
Osteoarthritis
Osteoarthritis of elbow
Osteoarthritis of hip
Polyarthritis (check for osteoarthritis)

Osteochondritis (OCD)
OCD - Osteochondritis dissecans
OCD - osteochondritis dissecans of hock
OCD - osteochondritis dissecans of stifle
OCD - osteochondritis dissecans of the shoulder
OCD - osteochondritis dissecans of the elbow (cases moved to elbow dysplasia)

Patella luxation
Dislocated patella

Subluxation of the patella

MALIGNANT NEOPLASIA
Adenocarcinoma
Adenocarcinoma
Adenocarcinoma of Anal Glands
Adenocarcinoma of bladder
Adenocarcinoma of lung
Adenocarcinoma of stomach
Ceruminous gland adenocarcinoma

Fibrosarcoma
Fibrosarcoma
Fibrosarcoma of spleen

Haemangiosarcoma
Angiosarcoma
Angiosarcoma of spleen
Haemangiosarcoma

Lymphosarcoma/lymphoma
Hodgkin's-like lymphoma of animals
Lymphosarcoma
Lymphoma
Lymphangiosarcoma
Pagetoid reticulosis

Mammary neoplasia
Malignant neoplasm of mammary gland
Neoplasm of mammary

MCT
Mastocytoma
Mast cell tumour

Melanocytic tumour
Limbal melanoma
Melanocytoma
Malignant melanoma
Malignant melanoma of ciliary body
Malignant melanoma of eye
Malignant melanoma of iris
Malignant melanoma of retina
Malignant melanoma of skin of ear

Osteosarcoma
Osteosarcoma
Osteochondrosarcoma

Squamous cell carcinoma
Squamous cell carcinoma

Transitional cell carcinoma
Transitional cell carcinoma of bladder

UROGENITAL
Urinary / reproductive tract tumours
Benign neoplasm of bladder
Benign neoplasm of vulva
Benign neoplasm of urethra
Benign neoplasm of uterus
Benign neoplasm of vagina
Malignant neoplasm of uterus
Malignant tumour of urethra
Malignant tumour of urinary bladder
Malignant tumour of vagina
Malignant tumour of vulva
Neoplasm of bladder
Neoplasm of vulva
Neoplasm of uterus
Neoplasm of urethra
Neoplasm of urinary tract
Neoplasm of urinary system
Neoplasm of vagina
Neoplasm of female genital organ
Sarcoma of bladder
Urinogenital tumour of uncertain behaviour

From above
Transitional cell carcinoma of bladder
Adenocarcinoma of bladder

Perivulvar dermatitis
Vulval dermatitis
Pyoderma (then had search for any around vulva)

Pseudopregnancy
False pregnancy

Pyometra
Pyometra

Recessed/inverted/juvenile vulva
Inverted vulva
Disorder of vulva (search for ‘inverted’ ‘recessed’ ‘juvenile’ only)

Struvite urolithiasis / urinary calculi
Urolithiasis
Urinary bladder stone
Kidney disease and search for ‘stone’ and ‘lithiasis’ ‘calculus’ ‘calculi’
Calculus in urethra

UI
Incontinence

Nocturnal incontinence of urine
Post-Micturition Incontinence
Urinary Incontinence

Urinary tract disorders
Dilatation of ureter
Disorder of the genitourinary system
Disorder of the urinary system
Disorder of ureter
Disorder of urethra
Disorder of urinary tract
Pyonephrosis
Ureteritis
Urethral Incompetence
Urethritis

USMI
Urinary incontinence due to urethral sphincter incompetence

UTI / cystitis
Cystitis
Haemorrhagic cystitis
UTI - Urinary tract infection

Vaginal / vulval disorder
Disorder of vagina (discharge cases moved to specific search)
Disorder of vulva (recessed, juvenile, inverted and discharge cases moved to specific search)
Vulval vestibulitis
Vulvitis
Vulval hyperplasia
Vaginal hyperplasia
Vaginal polyp

Vaginitis
Prepubertal vaginitis
Vaginitis
Vulvovaginitis

Vulval discharge, abnormal discharge
Abnormal vaginal discharge

IMMUNE
Immune mediated arthritis
Polyarthritis
Rheumatoid Arthritis

Otitis externa
Acute otitis externa
Chronic otitis externa
Otitis Externa

Atopy
Acute Atopic Conjunctivitis
Allergic skin disease
Atopic [allergic] Otitis
Atopic conjunctivitis
Atopic dermatitis

Autoimmune haemolytic anaemia
Autoimmune haemolytic anaemia
Haemolytic Anaemia (check for autoimmune otherwise no)

Hypoadrenocorticism
Addisons disease
Hypoadrenalism
Hypoadrenocorticism

Hypothyroidism
Hypothyroidism

Immune-mediated thrombocytopenia
Auto-immune thrombocytopenia
Thrombocytopenia (checked notes and included only those noted as immune mediated)

Inflammatory bowel disease (IBD)
Acute ulcerative colitis
Chronic Colitis (only if IBD)
Colitis (only if IBD)
Haemorrhagic Colitis (only if IBD)
Acute and Chronic colitis
Ulcerative Colitis (only if IBD)
Inflammatory bowel disease

Systemic lupus erythematosus
Systemic lupus erythematosus

OTHER
Aortic stenosis
Aortic stenosis

Overweight / obesity
BCS health codes 3,4,5,6,7,8,9
Overweight
Morbid obesity
Obese

Diabetes mellitus
Diabetes Mellitus

Early onset cataracts
Anterior capsular cataract
Anterior cortical cataract

Anterior subcapsular cataract
Bilateral cataracts
Cataract
Cortical cataract
Equatorial cataracts
Mature cataract
Nuclear cataract
Perinuclear cataract
Posterior capsular cataract
Posterior cortical cataract
Posterior polar capsular cataract
Posterior polar subcapsular cataract
Pulverulent cataract

Epilepsy (idiopathic)
Epilepsy (check for idiopathic)
Epileptic seizure (check for idiopathic)
Idiopathic generalised epilepsy
Status epilepticus (check for idiopathic)

Gastric volvulus
Gastric Dilatation-Volvulus-Torsion Syndrome

Geriatric cognitive impairment / CDS
Cognitive dysfunction
Senile dementia
Senile - old age
Senility

Histiocytoma
Benign fibrous histiocytoma of skin
Cutaneous histiocytoma
Dermatofibroma
Histiocytoma
