# Supplementary material for: A Prospective Study Investigating the Health Outcomes of Bitches Neutered Prepubertally or Post-Pubertally
Source: Animals (Basel). 2025 Jan 10;15(2):167. doi: 10.3390/ani15020167 (PMC11758339; doi:10.3390/ani15020167)
Supplement: Supplementary file 1 [file animals-15-00167-s001.zip › Animals Supplementary Materials 3 values of alpha revised.pdf]

Supplementary materials 3

Values of alpha for statistical analysis

Variable	N (PrePN, PostPN)	Alpha, Beta, effect size and sample size
Years of health data	155,151	4%, 5%, 0.249, 315
At least one disease from the group:	All=155,151	
Musculoskeletal		
Neoplasia		5%, 5%, 0.237, 232
Urogenital		4%, 5%, 0.316, 138
Immune disease		4%, 5%, 0.254, 213
Immune disease (excluding otitis)		4%, 5%, 0.204, 330
		4%, 5%, 0.344, 116
0, 1 or 2 diseases from the group:	All=155,151	
Musculoskeletal		4%, 5%, 0.255, 250
Urogenital		4%, 5%, 0.265, 230
Immune		5%, 5%, 0.218, 324
Disease incidence for:	All=155,151 unless stated	
Forelimb lameness		4%, 5%, 0.269, 190
Osteoarthritis		4%, 5%, 0.384, 93
Cruciate ligament		4%, 5%, 0.598, 39
Mast cell tumour		4%, 5%, 0.239, 241
Other neoplasia		4%, 5%, 0.314, 139
UTI/Cystitis		4%, 5%, 0.211, 309
Perivulval dermatitis		4%, 5%, 0.280, 175
Vulval/Abnormal discharge		4%, 5%, 0.223, 275
Otitis externa		4%, 5%, 0.208, 317
Atopy		4%, 5%, 0.211, 309
Histiocytoma		4%, 5%, 0.357, 108
Overweight/having obesity		5%, 5%, 0.201, 323
Obese based on BCS	90,89	5%. 5%, 0.270, 178
Age at first diagnosis for:		
Forelimb lameness**	29,25	10%, 5%, 0.910, 54
Osteoarthritis	18,11	9%, 5%, 1.345, 28
Mast cell tumour**	4,7	13%, 5%, 2.124, 11
Other neoplasia**	12,4	12%, 5%, 1.934, 16
UTI/Cystitis**	12,9	11%, 5%, 1.481, 21
Perivulval dermatitis**	8,5	13%, 5%, 1.905, 13
Vulval/Abnormal discharge**	5,8	13%, 5%, 1.905, 13
Atopy**	12,9	11%, 5%, 1.481, 21
Otitis externa**	74,55	10%, 5%, 0.589, 129
Overweight/having obesity**	65,69	10%, 5%, 0.572, 134
Musculoskeletal disease and growth†		

Cruciate ligament	7,218	9%, 5%, 0.396, 286
Forelimb lameness	40,185	9%, 5%, 0.397, 286
Osteoarthritis	19,206	9%, 5%, 0.400, 282
At least one musculoskeletal disease	59,166	9%, 5%, 0.394, 290
Urogenital disease and vulval appearance at 17 months†		
Perivulval dermatitis	13,265	4%, 5%, 0.494, 85
Urinary incontinence	7,271	4%, 5%, 0.580, 62
UTI/cystitis	20,258	4%, 5%, 0.438, 108
Vulval/abnormal discharge	13,265	4%, 5%, 0.494, 85
Urogenital disease and % dorsal fold coverage at 17 months†		
Perivulval dermatitis	7,158	4%, 5%, 0.611, 56
UTI/cystitis	11,154	4%, 5%, 0.439, 107
Vulval/abnormal discharge	10,155	4%, 5%, 0.396, 132
Urogenital disease and overall vulval growth from six to 17 months†		
Perivulval dermatitis	10,259	4%, 5%, 0.552, 68
Urinary incontinence	7,262	4%, 5%, 1.124, 17
UTI/cystitis	19,250	4%, 5%, 0.417, 119
Vulval/abnormal discharge	12,257	4%, 5%, 0.441, 107
Body condition score grouped as not obese/obese	90,89	5%, 5%, 0.270, 178
Overall health score	155,151	5%, 5%, 0.246, 308
Withdrawn from working**	97,98	5%, 5%, 0.281, 195
Withdrawn for health	44,53	4%, 5%, 0.380, 95
Number of bitches died	155,151	4%, 5%, 0.241, 236
Age at death**	18,14	10%, 5%, 1.200, 32

**Computed using sensitivity analysis not a priori.

†For comparisons between growth and development and bitches affected with disease, the number of bitches shown is for affected and unaffected, rather than PrePN and PostPN.
