# Supplementary material for: A Prospective Study Investigating the Health Outcomes of Bitches Neutered Prepubertally or Post-Pubertally
Source: Animals (Basel). 2025 Jan 10;15(2):167. doi: 10.3390/ani15020167 (PMC11758339; doi:10.3390/ani15020167)
Supplement: Supplementary file 1 [file animals-15-00167-s001.zip › Animals Supplementary Materials 4 Descriptive data for bitches diagnosed with each disease revised.pdf]

Supplementary materials 4 The number and percentage of bitches diagnosed with each disease along with descriptive data for age at diagnosis

Disease group	Disease	Trial group	Number bitches diagnosed (>6m of age)	% of bitches diagnosed	Number of cases	Mean +/- sem age diagnosis (years) (for all cases not just first)	Min and max age diagnosis (years) (for all cases not just first)
MSK	Cruciate rupture	All bitches	12	3.9	17	6.8 +/- 0.5	1.6 - 9.5
		PrePN	11	7.1	16	7.1 +/- 0.4	5.0 - 9.5
		PostPN	1	0.7	1	1.6 +/- 0.0	-
	ED	All bitches	6	2.0	6	3.2 +/- 1.2	0.6 - 8.3
		PrePN	3	1.9	3	1.6 +/- 0.7	0.6 - 3.0
		PostPN	3	2.0	3	4.8 +/- 2.1	1.1 - 8.3
	HD	All bitches	3	1.0	3	4.5 +/- 1.7	1.1 - 6.2
		PrePN	3	1.9	3	4.5 +/- 1.7	1.1 - 6.2
		PostPN	0	0.0	0	-	-
	Juvenile osteochondrosis	All bitches	0	0.0	0	-	-
		PrePN	0	0.0	0	-	-
		PostPN	0	0.0	0	-	-
	Osteoarthritis	All bitches	30	9.8	30	8.4 +/- 0.3	4.3 - 11.2
		PrePN	19	12.3	19	8.0 +/- 0.4	4.3 - 11.1
		PostPN	11	7.3	11	9.2 +/- 0.4	6.5 - 11.2
	OCD	All bitches	0	0.0	0	-	-
		PrePN	0	0.0	0	-	-
		PostPN	0	0.0	0	-	-
	Patella luxation	All bitches	1	0.3	1	7.7 +/- 0.0	-
		PrePN	1	0.6	1	7.7 +/- 0.0	-
		PostPN	0	0.0	0	-	-
	Forelimb lameness	All bitches	54	17.6	90	4.8 +/- 0.3	0.7 - 10.0
		PrePN	29	18.7	48	5.4 +/- 0.4	1.0 - 10.0
		PostPN	25	16.6	42	4.1 +/- 0.4	0.7 - 9.1
	MSk other	All bitches	4	1.3	4	7.4 +/- 0.8	6.0 - 9.5
		PrePN	3	1.9	3	6.6 +/- 0.4	6.0 - 7.3
		PostPN	1	0.7	1	9.5 +/- 0.0	-
Neoplasia	Adenocarcinoma	All bitches	0	0.0	0	-	-
		PrePN	0	0.0	0	-	-
		PostPN	0	0.0	0	-	-
	Fibrosarcoma	All bitches	0	0.0	0	-	-
		PrePN	0	0.0	0	-	-
		PostPN	0	0.0	0	-	-
	Haemangiosarcoma	All bitches	3	1.0	3	8.5 +/- 0.2	
		PrePN	1	0.6	1	8.6 +/- 0.0	-
		PostPN	2	1.3	2	8.4 +/- 0.4	8.0 - 8.8
	Lymphosarcoma/ lymphoma	All bitches	4	1.3	4	7.9 +/- 0.7	5.9 - 9.4
		PrePN	2	1.3	2	7.6 +/- 1.8	5.9 - 9.4
		PostPN	2	1.3	2	8.1 +/- 0.1	8.1 - 8.2
	Mammary neoplasia	All bitches	0	0.0	0	-	-
		PrePN	0	0.0	0	-	-
		PostPN	0	0.0	0	-	-
	MCT	All bitches	11	3.6	16	8.5 +/- 0.3	6.6 - 10.2
		PrePN	4	2.6	7	8.8 +/- 0.4	6.6 - 10.0
		PostPN	7	4.6	9	8.3 +/- 0.4	6.6 - 10.2
	Melanocytic tumour	All bitches	3	1.0	3	6.9 +/- 0.7	5.8 - 8.2
		PrePN	1	0.6	1	8.2 +/- 0.0	-
		PostPN	2	1.3	2	6.3 +/- 0.4	5.8 - 6.7
	Osteosarcoma	All bitches	0	0.0	0	-	-
		PrePN	0	0.0	0	-	-
		PostPN	0	0.0	0	-	-
	Squamous cell carcinoma	All bitches	1	0.3	1	9.6 +/- 0.0	-
		PrePN	1	0.6	1	9.6 +/- 0.0	-
		PostPN	0	0.0	0	-	-
	Transitional cell carcinoma	All bitches	0	0.0	0	-	-
		PrePN	0	0.0	0	-	-
		PostPN	0	0.0	0	-	-
	Neoplasia other	All bitches	16	5.2	17	8.0 +/- 0.6	3.1 - 11.1
		PrePN	12	7.7	13	8.0 +/- 0.8	3.1 - 11.1
		PostPN	4	2.6	4	8.1 +/- 0.3	7.2 - 8.7

Urogenital	Perivulvar dermatitis	All bitches	13	4.2	13	3.2 +/- 0.8	0.6 - 8.1
		PrePN	8	5.2	8	4.7 +/- 0.9	2.1 - 8.1
		PostPN	5	3.3	5	0.8 +/- 0.1	0.6 - 1.2
	Pseudopregnancy	All bitches	23	7.5	23	1.0 +/- 0.0	0.7 - 1.2
		PrePN	0	0.0	0	-	-
		PostPN	23	15.2	23	1.0 +/- 0.0	0.7 - 1.2
	Pyometra	All bitches	0	0.0	0	-	-
		PrePN	0	0.0	0	-	-
		PostPN	0	0.0	0	-	-
	Recessed/inverted/ juvenile vulva	All bitches	5	1.6	5	2.1 +/- 0.6	1.3 - 4.7
		PrePN	4	2.6	4	2.3 +/- 0.8	1.4 - 4.7
		PostPN	1	0.7	1	1.3 +/- 0.0	-
	Struvite urolithiasis / urinary calculi	All bitches	0	0.0	0	-	-
		PrePN	0	0.0	0	-	-
		PostPN	0	0.0	0	-	-
	UI	All bitches	7	2.3	8	4.8 +/- 0.8	2.4 - 7.7
		PrePN	1	0.6	1	7.7 +/- 0.0	-
		PostPN	6	4.0	7	4.4 +/- 0.8	2.4 - 7.2
	Urinary / reproductive tract tumours	All bitches	0	0.0	0	-	-
		PrePN	0	0.0	0	-	-
		PostPN	0	0.0	0	-	-
	Urinary tract disorders	All bitches	0	0.0	0	-	-
		PrePN	0	0.0	0	-	-
		PostPN	0	0.0	0	-	-
	USMI	All bitches	1	0.3	1	2.71 +/- 0.0	-
		PrePN	0	0.0	0	-	-
		PostPN	1	0.7	1	2.71 +/- 0.0	-
	UTI / cystitis	All bitches	21	6.9	27	3.1 +/- 0.6	0.5 - 11.1
		PrePN	12	7.7	15	2.6 +/- 0.9	0.5 - 11.1
		PostPN	9	6.0	12	3.7 +/- 0.8	0.6 - 7.2
	Vaginal / vulval disorder	All bitches	3	1.0	3	2.6 +/- 1.1	1.1 - 4.8
		PrePN	1	0.6	1	4.8 +/- 0.0	-
		PostPN	2	1.3	2	1.5 +/- 0.4	1.1 - 1.8
	Vaginitis	All bitches	3	1.0	3	0.5 +/- 0.0	0.5
		PrePN	0	0.0	0	-	-
		PostPN	3	2.0	3	0.5 +/- 0.0	0.5
	Vulval discharge, abnormal discharge	All bitches	13	4.2	13	2.0 +/- 0.7	0.5 - 7.7
		PrePN	5	3.2	5	2.6 +/- 1.3	0.5 - 7.6
		PostPN	8	5.3	8	1.7 +/- 0.9	0.5 - 7.7
Immune	Atopy	All bitches	21	6.9	21	2.9 +/- 0.4	0.8 - 6.7
		PrePN	12	7.7	12	3.9 +/- 0.6	1.0 - 6.7
		PostPN	9	6.0	9	1.6 +/- 0.3	0.8 - 3.7
	Autoimmune haemolytic anaemia	All bitches	1	0.3	1	6.9 +/- 0.0	-
		PrePN	1	0.6	1	6.9 +/- 0.0	-
		PostPN	0	0.0	0	-	-
	Hypoadrenocorticism	All bitches	0	0.0	0	-	-
		PrePN	0	0.0	0	-	-
		PostPN	0	0.0	0	-	-
	Hypothyroidism	All bitches	1	0.3	1	5.7 +/- 0.0	-
		PrePN	1	0.6	1	5.7 +/- 0.0	-
		PostPN	0	0.0	0	-	-
	Immune mediated arthritis	All bitches	2	0.7	2	2.9 +/- 1.7	1.2 - 4.5
		PrePN	2	1.3	2	2.9 +/- 1.7	1.2 - 4.5
		PostPN	0	0.0	0	-	-
	Immune-mediated thrombocytopenia	All bitches	1	0.3	1	2.9 +/- 0.0	-
		PrePN	1	0.6	1	2.9 +/- 0.0	-
		PostPN	0	0.0	0	-	-
	Inflammatory bowel disease (IBD)	All bitches	2	0.7	2	1.7 +/- 0.3	1.4 - 2.0
		PrePN	0	0.0	0	-	-
		PostPN	2	1.3	2	1.7 +/- 0.3	1.4 - 2.0
	Otitis externa	All bitches	129	42.2	255	2.8 +/- 0.1	0.5 - 10.4
		PrePN	74	47.7	145	2.7 +/- 0.2	0.5 - 10.4
		PostPN	55	36.4	110	2.9 +/- 0.2	0.5 - 8.8
	Systemic lupus erythematosus	All bitches	0	0.0	0	-	-
		PrePN	0	0.0	0	-	-
		PostPN	0	0.0	0	-	-

Other	Aortic stenosis	All bitches	2	0.7	2	0.8 +/- 0.3	0.6 - 1.2
		PrePN	0	0.0	0	-	-
		PostPN	2	1.3	2	0.8 +/- 0.3	0.6 - 1.2
	Diabetes mellitus	All bitches	1	0.3	1	9.6 +/- 0.0	-
		PrePN	0	0.0	0	-	-
		PostPN	1	0.7	1	9.6 +/- 0.0	-
	Early onset cataract	All bitches	4	1.3	4	1.2 +/- 0.1	1.1 - 1.3
		PrePN	2	1.3	2	1.2 +/- 0.1	1.1 - 1.3
		PostPN	2	1.3	2	1.2 +/- 0.1	1.2 - 1.3
	Any cataract	All bitches	8	2.6	8	4.3 +/- 1.3	1.1 - 10.2
		PrePN	4	2.6	4	5.1 +/- 2.3	1.1 - 10.2
		PostPN	4	2.6	4	3.5 +/- 1.5	1.3 - 7.7
	Epilepsy (idiopathic)	All bitches	2	0.7	2	4.8 +/- 2.4	2.4 - 7.2
		PrePN	2	1.3	2	4.8 +/- 2.4	2.4 - 7.2
		PostPN	0	0.0	0	-	-
	Gastric volvulus	All bitches	0	0.0	0	-	-
		PrePN	0	0.0	0	-	-
		PostPN	0	0.0	0	-	-
	Geriatric cognitive impairment / CDS	All bitches	0	0.0	0	-	-
		PrePN	0	0.0	0	-	-
		PostPN	0	0.0	0	-	-
	Histiocytoma	All bitches	10	3.3	10	1.6 +/- 0.2	0.6 - 2.8
		PrePN	7	4.5	7	1.6 +/- 0.2	0.8 - 2.5
		PostPN	3	2.0	3	1.6 +/- 0.6	0.6 - 2.8
	Overweight/obese	All bitches	134	43.8	134	3.2 +/- 0.2	0.7 - 11.3
		PrePN	65	41.9	65	3.4 +/- 0.3	0.8 - 11.3
		PostPN	69	45.7	69	2.9 +/- 0.3	0.7 - 9.4
	Obesity (based on BCS 7+)	All bitches	44	14.4	44	5.4 +/- 0.3	2.0 - 10.6
		PrePN	20	12.9	20	5.1 +/- 0.4	2.0 - 8.3
		PostPN	24	15.9	24	5.7 +/- 0.5	2.5 - 10.6
